# Social phobia in childhood-onset systemic lupus erythematosus

**DOI:** 10.1186/1546-0096-12-S1-P333

**Published:** 2014-09-17

**Authors:** Catia Nascimento, Renata Barbosa, Luciana Oliveira, Karina Peliçari, Nailu Sinicato, Roberto Marini, Paula Fernandes, Simone Appenzeller

**Affiliations:** 1 Medicine, State University Of Campinas, Campinas, Brazil; 2 Pediatrics, State University Of Campinas, Campinas, Brazil; 3 Sports Medicine, State University Of Campinas, Campinas, Brazil

## Introduction

Anxiety is frequently observed in systemic lupus erythematosus, however the prevalence of phobia has rarely been reported.

## Objectives

To evaluate the frequency of social phobia in patients with childhood-onset Systemic Lupus Erythematosus (cSLE) and to verify possible associations with clinical, laboratory manifestations, and the use of corticosteroids.

## Methods

In this cross-sectional study 48 patients with cSLE followed at the Pediatric Rheumatology Unit of the State University of Campinas during the May and August of 2013 were included. The control group was composed by 53 age and sex matched controls. The presence of symptoms of social phobia was assessed by the Liebowitz Social Anxiety Scale (LSAS), validated in portugues. Neuropsychiatric manifestations were classified according to the criteria established by the American College of Rheumatology. SLE patients were further assessed for clinical and laboratory SLE manifestations, disease activity [SLE Disease Activity Index (SLEDAI)], damage [Systemic Lupus International Collaborating Clinics/American College of Rheumatology Damage Index (SDI)] and current drug exposures. Total dose of corticosteroids and other immunosuppressant medications used since the onset of disease were calculated by data obtained by careful review of the medical charts.

## Results

Social phobia was observed in 9 (18.75%) patients and was associated with the current age (p = 0.003), age at diagnosis (p = 0.008), disease duration (p = 0.007), SLEDAI (p = 0.009), cumulative corticosteroid dose adjusted by weight (p = 0.002), current dose (p = 0.004) and the cumulative dose over time (p = 0.001). When analyzed by severity, moderate social phobia was observed in 5 (10.42%) patients and associated with disease duration (p = 0.005), SLEDAI adjusted over time (p = 0.02), headaches (p = 0.048) and cumulative dose of corticosteroids (p = 0.009). Severe social phobia was observed in 3 (6.25%) patients and was associated with current age (p = 0.035). Very severe social phobia was observed in one (2.08%) patient and associated with SLEDAI (p = 0.036). There was no significant association between social phobia and cumulative damage and other neuropsychiatric manifestations.

## Conclusion

Social phobia was frequently observed in cSLE and associated with disease activity and corticosteroid dosage. Social phobia should be screened routinely it can influence the quality of life of cSLE patients.

## Disclosure of interest

None declared

